# Overexpression of *OsDof12* affects plant architecture in rice (*Oryza sativa* L.)

**DOI:** 10.3389/fpls.2015.00833

**Published:** 2015-10-08

**Authors:** Qi Wu, Dayong Li, Dejun Li, Xue Liu, Xianfeng Zhao, Xiaobing Li, Shigui Li, Lihuang Zhu

**Affiliations:** ^1^Rice Research Institute, Sichuan Agricultural UniversityChengdu, China; ^2^State Key Laboratory of Plant Genomics and National Center for Plant Gene Research, Institute of Genetics and Developmental Biology, Chinese Academy of SciencesBeijing, China; ^3^Key Laboratory of Biology and Genetic Resources of Rubber Tree, Rubber Research Institute, Chinese Academy of Tropical Agricultural SciencesDanzhou, China; ^4^Key Laboratory of Genome Sciences and Information, Beijing Institute of Genomics, Chinese Academy of SciencesBeijing, China

**Keywords:** *OsDof12*, Dof transcription factor, plant architecture, rice (*Oryza sativa* L.)

## Abstract

Dof (DNA binding with one finger) proteins, a class of plant-specific transcription factors, are involved in plant growth and developmental processes and stress responses. However, their biological functions remain to be elucidated, especially in rice (*Oryza sativa* L.). Previously, we have reported that *OsDof12* can promote rice flowering under long-day conditions. Here, we further investigated the other important agronomical traits of the transgenic plants overexpressing *OsDof12* and found that overexpressing *OsDof12* could lead to reduced plant height, erected leaf, shortened leaf blade, and smaller panicle resulted from decreased primary and secondary branches number. These results implied that *OsDof12* is involved in rice plant architecture formation. Furthermore, we performed a series of Brassinosteroid (BR)-responsive tests and found that overexpression of *OsDof12* could also result in BR hyposensitivity. Of note, in WT plants the expression of *OsDof12* was found up-regulated by BR treatment while in *OsDof12* overexpression plants two positive BR signaling regulators, *OsBRI1* and *OsBZR1*, were significantly down-regulated, indicating that *OsDof12* may act as a negative BR regulator in rice. Taken together, our results suggested that overexpression of *OsDof12* could lead to altered plant architecture by suppressing BR signaling. Thus, *OsDof12* might be used as a new potential genetic regulator for future rice molecular breeding.

## Introduction

DOF (DNA binding with one finger) proteins are plant-specific transcription factors (Yanagisawa, 2002; Moreno-Risueno et al., 2007; Noguero et al., 2013). The Dof domain consists of 52 amino acid residues encompassing the CX_2_CX_21_CX_2_C motif (Yanagisawa, 2002; Umemura et al., 2004). Dof transcription factors, with the exception in pumpkin, usually regulate the expression of the target genes *via* binding a core DNA motif with an essential sequence of AAAG (Yanagisawa and Schmidt, 1999). Dof proteins are widespread and versatile regulators for various biological processes such as metabolism regulation, phytohormone response, seed germination and development, photoperiodic flowering and plant patterning in plants (Yanagisawa, 2002; Noguero et al., 2013). In carbohydrate metabolism, *ZmDof1* and *ZmDof2* acted antagonistically to control the expression of *C4 phosphoenolpyruvate carboxylase* (*PEPC*) in maize (*Zea mays*) (Yanagisawa, 2000). In Arabidopsis (*Arabidopsis thaliana*), *AtDof1.1*/*OBP2* participated in regulation of indole glucosinolate biosynthesis (Skirycz et al., 2006). Overexpression of *OsDof25* in Arabidopsis changed the nitrogen and carbon metabolism (Santos et al., 2012). In tobacco (*Nicotiana tabacum*), NtBBF1 (roiB domain B Factor) can bind to the *rolB* promotor in an auxin-regulated way to modulate its expression, which betters our understanding the mechanism underlying auxin induction (Baumann et al., 1999). Besides, several cases have documented that *Dof* genes are implicated in seed germination and development. The *DOF gene Affecting Germination-1* (*DAG1*) and *DOF gene Affecting Germination-2* (*DAG2*) controlled seed germination in Arabidopsis *via* a maternal switch (Papi et al., 2000; Gualberti et al., 2002). In rice, *OsDof3* might enhance expression of *type3 carboxypeptidase* (*CPD*) under GA control in aleurones (Washio, 2001), and further investigation indicated that OsDof3 interacts with GAMYB to regulate synergically the expression of *RAmy1A* to mediate GA signaling during rice seed germination (Washio, 2003). RPBF (rice prolamin box binding factor) interplayed with the rice basic leucine zipper factor RISBZ1 to maintain proper expression of rice seed storage protein genes during seed development (Kawakatsu et al., 2009).

Moreover, Dof factors are involved in photoperiod flowering. In *Arabidopsis*, Cycling Dof Factor-1 (CDF1) binds to the *COSTANS* (*CO*) and *FLOWERING LOCUS T* (*FT*) promotor regions to block transactivation of this two flowering genes, whereas this inhibition could be released based on the GIGANTEA-FLAVIN-BINDING, KELCH REPEAT, F-BOX1(GI-FKF1) complex mediated degradation of CDF1 under long-day conditions (Imaizumi et al., 2005; Sawa et al., 2007; Song et al., 2012). Fornara et al. (2009) systematically studied a subset of *Dof* family related to *CDF1* and found that *CDF1, CDF2, CDF3*, and *CDF5* acted redundantly to repress flowering by decreasing the mRNA level of *CO* (Fornara et al., 2009). In rice, *RDD1* (*rice Dof daily fluctuations 1*) was controlled by circadian clock, and repressing the expression of *RDD1* led to delayed flowering time (Iwamoto et al., 2009). Furthermore, several studies have revealed the importance of Dof factors on plant patterning. In Arabidopsis, AtDof5.1 modulated leaf adaxial–abaxial polarity *via* binding to the promotor of *Revoluta* (*REV*) and enhancing expression of *REV* (Kim et al., 2010). *AtDOF4.2* and *AtDOF4.4* were engaged in regulating shoot branching and seed development (Skirycz et al., 2007; Zou et al., 2013). *OBF-binding factor-1*(*OBP1*) is a cell cycle regulator, and overexpressing *OBP1* in Arabidopsis affects cell size and number, rendering dwarfish plant morphology (Skirycz et al., 2008).

To now, of the 36 predicted *Dof* homologous genes in whole Arabidopsis genome, 16 have been confirmed to participate in various biological processes (Noguero et al., 2013). However, in rice, to our knowledge, the studies deciphering Dof factors are rather limited; only 5 of the 30 predicted *Dof* members have been characterized in detail (Washio, 2003; Yamamoto et al., 2006; Li et al., 2008, 2009b; Iwamoto et al., 2009; Santos et al., 2012). In a previous study, we characterized the function of *OsDof12* in rice (Li et al., 2008, 2009b). We found a pair of sense-antisense transcript at the locus of *OsDof12* (LOC_Os03g07360), denoted as *OsDof12* (sense transcript) and *OsDof12os* (antisense transcript), respectively. *OsDof12* encodes a nuclear-localized protein of 440 amino acids (Li et al., 2008). Expression pattern analysis showed *OsDof12* and *OsDof12os* were co-expressed but reciprocally regulated by each other (Li et al., 2008). Moreover, overexpression of *OsDof12* promoted flowering in rice under long-day conditions by up-regulating rice florigen-encoding gene *Hd3a* and its downstream gene *OsMADS14* (Li et al., 2009b). In wild type rice plants, the transcripts of *OsDof12* were detected in various rice tissues at different development stages (Li et al., 2009b), which suggests *OsDof12* might take part in various biological processes.

In current study, we demonstrated that the transgenic plants overexpressing *OsDof12* indeed display pleiotropic phenotypes such as reduced plant height, shortened leaf length, more erected leaf, smaller panicle size and decreased grain yield. Further molecular biological analyses indicated that these changes in plant architecture could be ascribed to attenuated BR signaling in *OsDof12* overexpression plants.

## Materials and methods

### Plant materials and growth conditions

To observe the influence of *OsDof12* on plant architecture, the wild type cultivar Nipponbare (*Oryza sativa* L. ssp. *japonica* cv. Nipponbare) and the previous reported two *OsDof12* overexpressing lines (Line OD2 and Line OD5, Nipponbare background) (Li et al., 2009b) were grown on the research field located in the Experimental Stations of the Institute of Genetics and Developmental Biology, Chinese Academy of Sciences, Beijing, under nature field conditions. After mature stage (approximately 120 days after sowing), we measured the agronomic traits including plant height, internode length, leaf length, and panicle architecture.

For BR sensitivity tests, plants were grown on 1/2 Murashige and Skoog (MS) culture medium in a growth chamber under controlled conditions (16h/light, 30°C and 8h/dark, 26°C) for 7 or 8 days. For skotomorphogenesis analysis, the WT and OD plants were grown on 1/2 MS at 28°C in darkness for 2 weeks. For *OsDof12* induction analysis, 2-week-old WT plants grown on 96-well PCR plates were treated with 100 nM Brassinolide (BL, the most bioactive BR compound, not being synthesized in rice, Yokota, 1997; Fujioka and Yokota, 2003; Kim et al., 2008; Vriet et al., 2013) solution, then the hole plants were sampled at different time points for expression assay. For expression levels analysis on BR related genes, 7-day-old plants of WT and OD were used.

### Microscopic observation

The middle part of the second internode from mature plants were harvested and fixed with 2.5% glutaraldehyde solution overnight at 4°C, and then dehydrated in order by 40, 50, 60, 70, 80, 90, 95, and 100% ethanol solutions. The samples got dried through critical point drying by liquid carbon dioxide. The dry specimens were mounted on a stub, gold-coated with an ion sputter coater (Hitachi, Tokyo, Japan) and then imaged with a scanning electron microscope (Hitachi, Tokyo, Japan).

### BR treatment

The seeds were dehusked and sterilized with 75% ethanol for 1 min, 3% NaClO for 25 min and then washed five times with sterile water. The seeds were grown on solid 1/2 MS medium containing a series of concentration of BL (WAKO, Japan) for 7 or 8 days. The lengths of second leaf sheath, second leaf, seedling height, root, and coleoptile were measured for further analyses.

### BR measurement

After sterilization, the seeds of WT and OD were sown and grown on 1/2 MS culture medium for 10 days, then about 1 gram of fresh seedlings were collected for measurement of Castasterone (CS, one of the biologically active BR compounds, a likely end product of brassinosteroid biosynthetic pathway in rice, Kim et al., 2008; Vriet et al., 2013) according to the method described previously (Ding et al., 2013).

### Laminal inclination assay

The seeds were immersed for 2 days, and then the germinated seeds were sown on 1/2 MS supplied with different concentrations of BL. After incubation in a growth chamber for 8 days, the second lamina joints on WT and OD plants were imaged and measured by IMAGEJ according to the method described by Tong et al. (2009).

### RNA extraction and qRT-PCR analysis

The samples were harvested and stored in liquid nitrogen until RNA extraction. The isolation of total RNA was performed applying Trizol reagent (Invitrogen, California, USA) with corresponding protocol. DNA digestion was accomplished by DnaseI (Takara, Japan), and first-strand cDNA was obtained by GoScript Reverse Transcription System (Promega, http://www.promega.com/). With a rice ubiquitin gene (*UBQ, LOC_Os03g13170*) set as the internal control, qRT-PCR was performed using EvaGreenq PCR MasterMix (Abm, Canada) on a real-time PCR System (Bio-Rad CFX96) with the specific primers listed in Table S2. The qRT-PCR program consists of 95°C for 3 min and 42 cycles of 95°C for 5 s, 60°C for 10 s. The relative expression level of each examined gene was quantified by a relative quantization method.

## Results

### Overexpression of *OsDof12* reduces plant height

**Figure 1:**
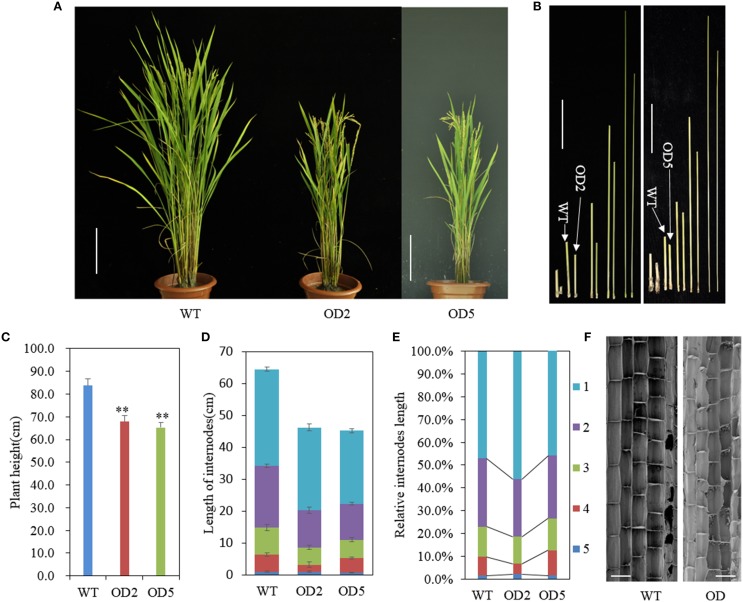
*****OsDof12*** overexpression plants showed dwarf phenotype. (A)** Gross morphology comparison between wild type (WT) and *OsDof12* overexpressing transgenic plants (OD) at maturity stage (approximately 120 days after sowing). The OD plants showed significantly reduced plant height and more compacted plant architecture than WT plants. Bar = 15 cm. **(B)** Comparison of the internode length. Left: WT vs. OD2; Right: WT vs. OD5. Bar = 5 cm. **(C)** Statistical analysis of plant height. Values are mean ± sd (*n* = 30). Double asterisks (^**^) stands for *P* < 0.01 determined by student's *t*-test. **(D)** Schematic representation of each individual internode length. Values are mean ± sd, *n* = 15. **(E)** The percentage of each internode length accounting for the total culm length. **(F)** Microscopic observation by SEM on the longitudinal section of the middle part in the second internode. Bar = 100μm.

**Figure 2:**
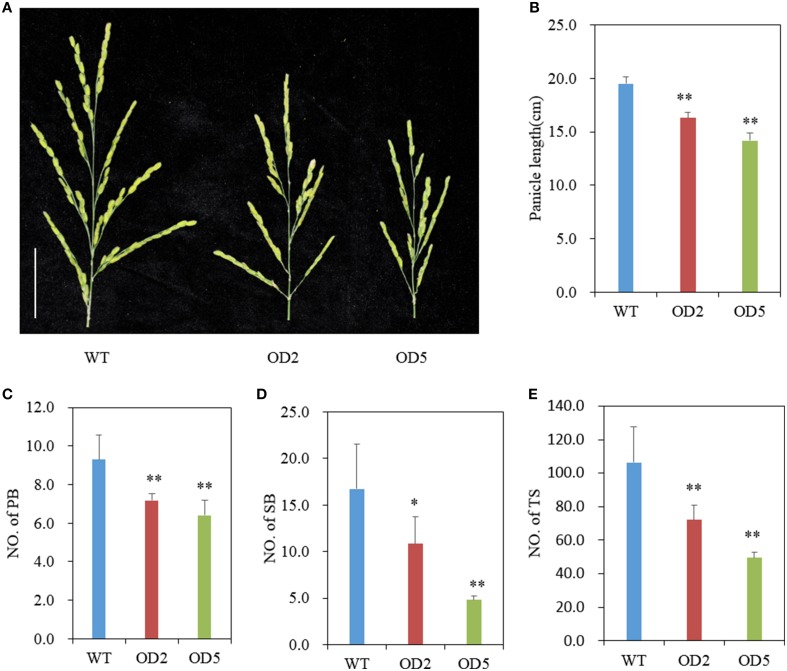
**Comparison of panicle structure. (A)** Panicle morphology comparison between WT and OD plants. Bar = 5 cm. **(B-E)** Statistical results of panicle length **(B)**, number of primary branches **(C)**, secondary branches **(D)**, and total spikelets per panicle **(E)** in WT and *OsDof12* overexpression plants. PB, primary branches; SB, secondary branches; TS, total spikelets. Values are mean ± sd (*n* = 30). Single asterisk (^*^) and double asterisks (^**^) stand for *P* < 0.05 and *P* < 0.01, respectively, determined by student's *t*-test.

In our previous study, we developed *OsDof12* overexpressing lines in rice (hereafter named OD) (Li et al., 2009b). The OD lines, OD2 and OD5, and WT plants were grown in a paddy field in Beijing (40°10′N, 116°42′E) under nature field conditions. In order to investigate the effects of *OsDof12* overexpression on rice agronomic traits, we traced the growth performance of OD and WT plants and found that the plant height of OD was almost the same as WT before heading stage. However, after the plant height was stable at mature stage, on average, the WT plant height reached approximate 83.7 cm while OD plant height was about 65.3 cm and 20% shorter than the WT (Figures 1A,C). Rice plant height consists of the internodes length and the panicle length. We measured and compared the internode and panicle lengths of OD plants with those of WT plants, respectively. Statistic data showed that the individual internode and panicle lengths in OD were remarkably shorter than those in WT (Figures 1B,D, 2B), which consequently led to decreased plant height of OD plant. Comparing the percentage length of each internode to the total culm in OD with that in WT, we found that the second and fourth internode lengths of OD were largely and slightly reduced (Figure 1E), respectively.

Internode elongation is determined by cell division activity in the intercalary meristem, followed by cell elongation in the elongation zone (Yamamuro et al., 2000). To investigate whether those two factors caused the dwarfish morphology of OD, we observed the longitudinal cell morphology of the OD and WT internodes. After heading stage, we collected and fixed the middle sections of the second internodes, then observed the cell length under scanning electron microscope (SEM). As shown in Figure 1F, there was no obvious difference in the longitudinal cell length between WT and OD, thus the reduction in longitudinal cell number on the elongation zone may account for the shortage of the internodes in OD.

### *OsDof12* overexpression plants produce smaller panicles

Besides plant height, we also analyzed the panicle structure and seed size in OD plants. As shown in Figures 2A,B, the panicles of OD are shorter and smaller than that of WT. We further investigated the panicle index including numbers of primary branches, secondary branches, and total spikelets, and found that the number of primary branches in OD was remarkably reduced (Figure 2C), meanwhile OD plants produced significantly less secondary branches (Figure 2D). And, expectedly, there was very significant reduction in total spikelet number per panicle in OD as compared with that in WT (Figure 2E). Nevertheless, no obvious differences of seed size and weight appeared between OD and wild type (Figure S1). These results imply that overexpression of *OsDof12* could lead to smaller panicle structure with reduced primary branches, secondary branches and total spikelets.

### Abnormal leaf morphology in *OsDof12* overexpression plants

**Figure 3:**
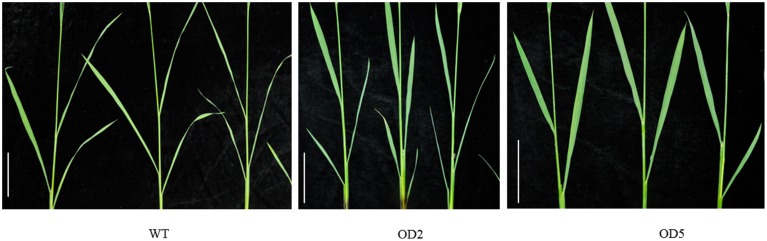
**Comparison of the lamina joint zones**. The leaf angle in OD2 and OD5 plants is less enlarged than WT plants. Bar = 10 cm.

Aside from the alternations on plant height and panicle structure, OD also exhibited some other characteristic phenotypes. In comparison with WT plants, OD plants generated more compacted plant architecture with more erected leaves (Figure 1A). As shown in Figure 3, obviously the leaf joint angle on OD plants was smaller than that on WT plants. Moreover, we measured the length of flag leaves, penultimate leaves and antepenultimate leaves of OD and WT plants. Statistical analysis indicated that the leaves in OD plants were remarkably shorter than those in WT plants (Figure S2).

### Overexpression of *OsDof12* altered BR sensitivity

Brassinosteroids (BRs) play pivotal roles in regulating plant growth and development (Müssig and Altmann, 2001; Fujioka and Yokota, 2003; Salas Fernandez et al., 2009; Tong and Chu, 2012; Wang et al., 2012; Zhu et al., 2013). By now, many BR metabolic and signaling-related genes have been well characterized (Yamamuro et al., 2000; Hong et al., 2002, 2003, 2005; Bai et al., 2007; Tanaka et al., 2009; Tong et al., 2009, 2012; Li et al., 2009a; Sakamoto et al., 2011; Thornton et al., 2011; Zhang et al., 2012). Notably, almost all of the corresponding mutants or misexpressors display abnormal plant height and leaf inclination phenotypes. Considering that the plants overexpressing *OsDof12* exhibit reduced plant height and erect leaf morphology, we assumed that *OsDof12* might be involved in BR metabolism or signal transduction. To test this hypothesis, we designed and conducted a series of experiments to evaluate the BR response of OD2 plants.

Firstly, we measured the endogenous BR levels by quantifying the CS content in WT and OD2 plants, respectively. As a matter of fact, no obvious difference in BR levels between WT and OD plants was observed (Table S1), suggesting that *OsDof12* overexpression may not affect the metabolism of BRs, which was further confirmed by expression level analysis on BR metabolism related genes (Figure S3).

**Figure 4:**
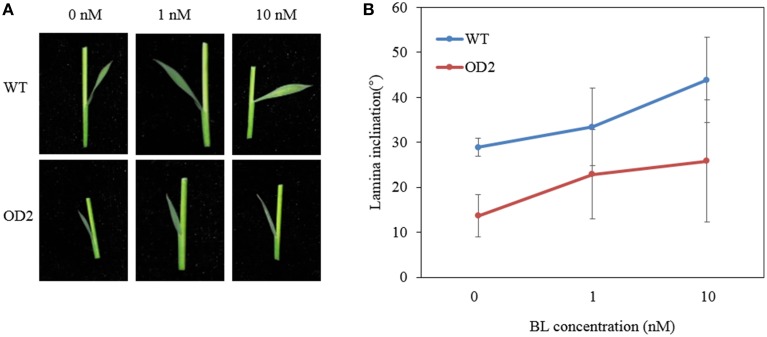
**Lamina joint bending analysis. (A)** The second lamina joint morphology in WT and OD2 plants grown on BL contained half-strength MS for 7 days. **(B)** Quantification of the second lamina angel bending as shown in **(A)**, values are mean ± sd, *n* = 20.

Then, we performed lamina joint bending assay by applying BL on the plants. Notably, the lamina joints of OD plants were remarkably less enlarged than that of WT when both subjected to a mock treatment without BL (Figure 4A). When treated with the increasing doses of BL, though the angles of lamina joint on both OD and WT plants became relatively larger, the lamina inclination curve of OD ascended much slower than that of WT (Figure 4B), indicating that overexpression of *OsDof12* may cause defects on the BR signal transduction pathway leading to impaired bending of leaves.

**Figure 5:**
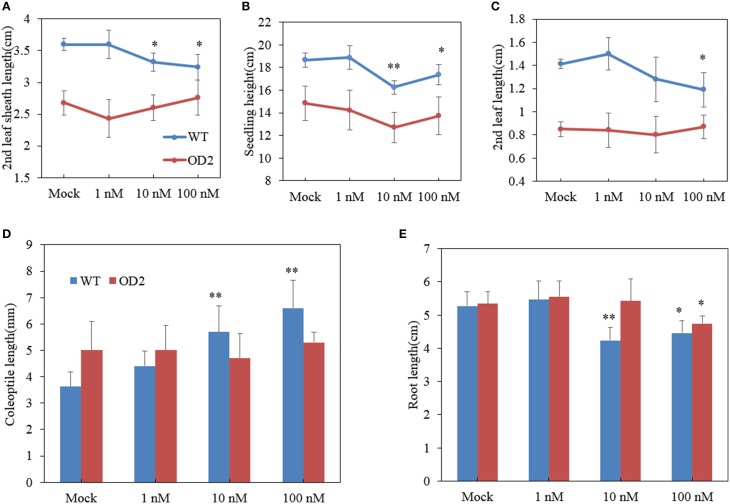
**Altered BR sensitivity in overexpressing transgenic plants**. **(A–E)** Statistical analysis of the 2nd leaf sheath length **(A)**, seedling height **(B)**, 2nd leaf length **(C)**, coleoptile length **(D)**, and root length **(E)** in plants treated by various levels of BL. Values are mean ± sd, *n* = 15. Single asterisk and double asterisks respectively stand for *P* < 0.05 and *P* < 0.01 determined by student's *t*-test.

Besides OD plants exhibiting lamina joint abnormality, we also found that overexpression of *OsDof12* affected other aspects of BR-related morphology. For instance, the second leaf sheath length of OD plants was apparently shorter than that of WT plants. In WT plants, when treated with 1 nM BL, the second leaf sheath length slightly but not significantly increased than those in the mock treatment, however, 10 nM or higher concentrations of BL treatments gave rise to evidently shorter second leaf sheath length in WT plants (Figure 5A), suggesting that relative higher level of exogenous BR inhibits the elongation of the leaf sheath. In contrast, various concentrations of BL treatment on OD could hardly altered the elongation of the second leaf sheath (Figure 5A), indicating that the elongation of the second leaf sheath in OD was hyposensitive to exogenous BR treatment. The similar situation also happened to the elongation of the second leaf and seedling height in WT and OD plants, respectively. The second leaf length and seedling height in OD plants was apparently shorter than those in WT. Relative higher concentrations of BL decreased evidently the elongation of the second leaf and seedling height in WT plants (Figures 5B,C), while various levels of BL treatment could hardly affect the elongation of the second leaf length and seedling height in OD plants (Figures 5B,C), indicating OD plants may have lower sensitivity in response to exogenous BR.

### Elongation of the coleoptiles and root in response to BL

The coleoptile length and root elongation are respects to evaluate BR sensitivity of plants (Yamamuro et al., 2000). Thus, we measured the coleoptile length of 8-day-old seedlings. As shown in Figure 5D, the coleoptile elongation of WT was promoted by BL treatment in dose-dependent manner. However, the coleoptile elongation of OD plants showed no difference between BL and mock treatment.

Similar root growth pattern was shared by OD and WT plants when grown in 0 nM or 1 nM BL medium, 1 nM BL slightly increased the root elongation (Figure 5E). When treated with 10 nM or 100 nM BL, the WT plants generated apparently shorter root. However, for OD plants, 10 nM BL could not obviously reduce root elongation, only up to 100 nM BL treatment could inhibit the root elongation (Figure 5E). The results also suggested that overexpression of *OsDof12* may result in lower BR sensitivity.

### Skotomorphogenic phenotypes of *OsDof12* overexpressing plants

**Figure 6:**
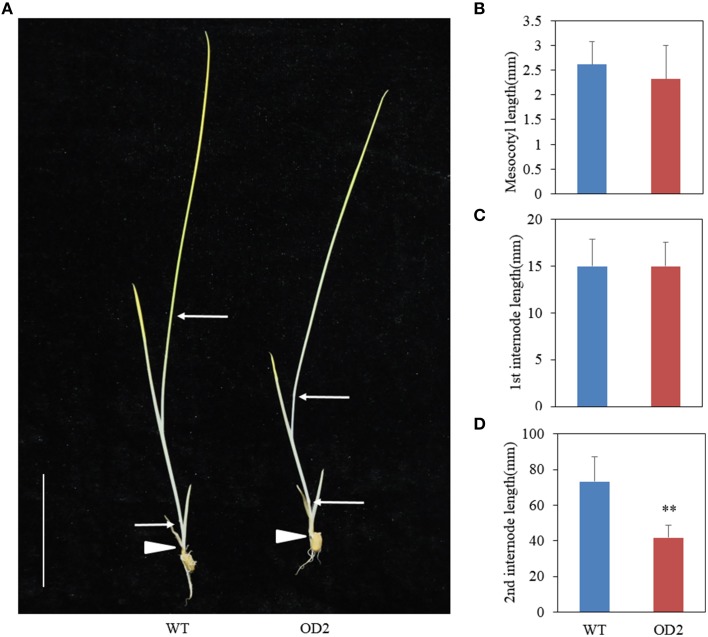
**Skotomorphogy analysis. (A)** Two-week-old seedlings grown on half-strength MS in dark conditions. The mesocotyl **(B)** and first internode **(C)** length of OD2 is comparable to WT, whereas the second internode **(D)** was obviously shortened in OD2.Values are mean ± sd, *n* = 12. Double asterisks stand for *P* < 0.01 determined by student's *t*-test. Arrows and arrowheads indicate the nodes and mesocotyls, respectively. Bar = 25 mm.

The phenotypes of mesocotyl and internode elongation in darkness have been employed as a good criteria to determine whether the dwarf phenotype is related to BR (Hong et al., 2002, 2003). To test whether overexpression of *OsDof12* affects the elongation of mesocotyl and internode, we grew the seeds on half-strength MS under totally dark conditions. Two weeks later, we observed the phenotypes of WT and OD plants and found that the elongation of mesocotyl and first internode in OD plants was similar to those in WT plants (Figures 6A–C), however, the second internode length in OD plants was much less than that in WT plants (Figures 6A,D), indicating overexpression of *OsDof12* specifically inhibits the elongation of the internode in dark conditions.

### Expression patterns of BR signaling related genes were changed in transgenic plants overexpressing *OsDof12*

**Figure 7:**
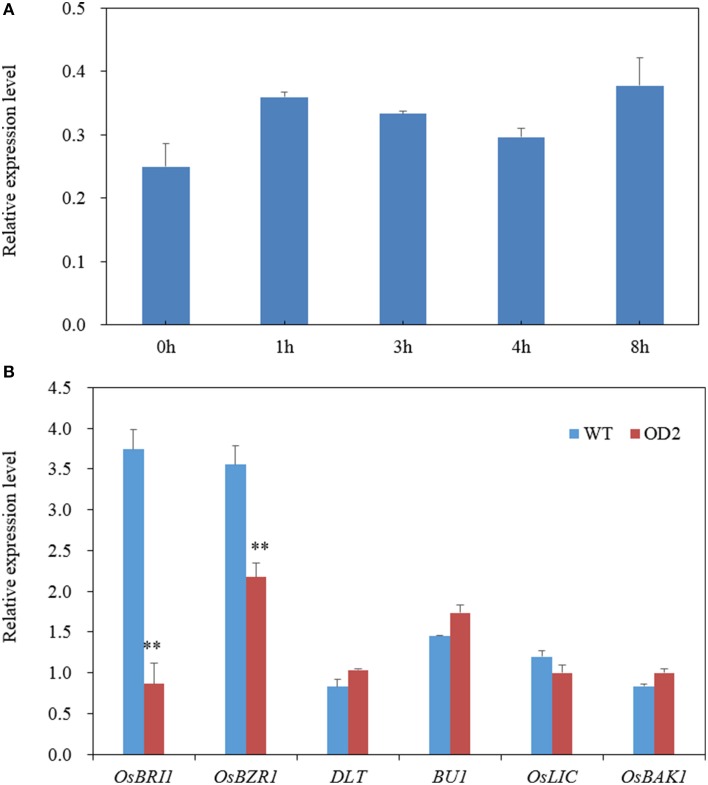
**Gene expression analysis. (A)** *OsDof12* induction analysis. Two-week-old WT seedlings were subjected to exogenous 100 nM BL treatment, then whole plants were collected at different time point for gene expression analysis. **(B)** qRT-PCR analysis of BR signaling genes. Seven-day-old seedlings grown on half-strength MS were used for expression analysis. Values are mean ± sd, *n* = 3. Double asterisks stand for *P* < 0.01 determined by student's *t*-test.

As demonstrated above, *OsDof12* may be a regulator for maintaining normal BR signaling. We then test whether *OsDof12* responds to BR treatment. As shown in Figure 7A, the *OsDof12* transcripts rapidly accumulated after BL treatment for 1 h; although *OsDof12* gradually descended from 2 to 4 h, it was still higher than that before BR treatment; after BR treatment the transcriptional level peak of *OsDof12* appeared at 8 h. This result suggests *OsDof12* could be induced by exogenous BR, which indicates *OsDof12* might be involved in BR regulation.

Using qRT-PCR, we further analyzed the expression level of six major BR signaling components. As shown in Figure 7B, *OsBRI1* and *OsBZR1* were down-regulated, while *OsBU1, OsLIC*, and *DLT* were not affected, suggesting that *OsDof12* is possibly involved in BR signaling pathway.

## Discussion

*Dof* genes have been known involved in various processes at different development stages in plants (Yanagisawa, 2002; Noguero et al., 2013). In previous study, we focused on overexpression of *OsDof12* promoting flowering in rice under long-day conditions. In the present study, we demonstrate that overexpression of *OsDof12* can alter the rice plant architecture. Further evidences indicate *OsDof12* may participate in BR signaling to modulate rice architecture. All these clearly show that *OsDof12* is a pleiotropic regulator for plant growth and development.

Higher plants have developed a complex of metabolic mechanisms, which include biosynthetic and catabolic pathway, to maintain BR homeostasis for normal growth and development (Tanaka et al., 2005; Vriet et al., 2013). The abnormal phenotypes in OD plants are similar with the phenotypes of BR-deficient mutants. Therefore, we investigated this possible clues that *OsDof12* might be involved in BR metabolism. Measurement of the endogenous BR levels indicated that the BR content in plants overexpressing *OsDof12* was not affected. Meanwhile, we quantified expression levels of BR metabolic genes, such as *D2, D11, OsDWARF, OsDWARF1, OsDWARF4, CYP734A*s and no obvious difference were found between OD and WT plants. These results excluded the possibility that *OsDof12* might modulate plant architecture by affecting BR metabolism pathway.

Notably, a range of BR response tests on *OsDof12* overexpression plants, including lamina joint assay, sheath, root, coleoptile elongation pattern analysis, and skotomorphogenesis analysis all suggest that overexpression of *OsDof12* reduces the BR sensitivity. Besides, the *OsDof12* expression was induced by exogenous BL treatment and the expression patterns of two BR signaling components were suppressed in *OsDof12* overexpression plants, which further suggest that *OsDof12* is involved in coordinating rice BR signaling.

Rice plant architecture is composed of tiller number, internode elongation, leaf angle and panicle structure, and favorable architecture is able to facilitate improving the yield (Wang and Li, 2006, 2008; Yang and Hwa, 2008). Plant height is one of the most important agronomic traits (Sakamoto and Matsuoka, 2004; Wang and Li, 2008). Rice dwarf phenotypes have been well categorized into several patterns (Yamamuro et al., 2000), and BR-associated mutants usually display dm-type dwarfism where the second internode is specifically shortened. The *OsDof12* overexpression lines, OD2 and OD5, both generated shortened internodes, among which the relative length of the second internode were shortened significantly, therefore, we speculate that they belong to dm-type dwarfism. As suggested by our microscopic observation in the elongation zone, being different from the two BR mutants, *d61-2* (Yamamuro et al., 2000) and *dlt* (Tong et al., 2009) where the elongation of longitudinal cell is severely hampered, the cell length in OD plants is not affected, which might indicate that the cell number decrease should be the major cause for the dwarfism in OD. These results suggest that the regulatory mechanism underlying cell elongation and plant height appear to be quite complicated, thus the detail mechanism in *OsDOf12* regulation of plant height should be further studied.

Erect leaf pattern is another desirable trait for ideal plant architecture. Generally, the erect leaves in dense plantings allow more light penetrating through the upper leaves layer to make the lower leaves layer capture more light for photosynthesis and assimilation (Sinclair and Sheehy, 1999). Indeed, several cases have witnessed the effect of erect leaf on improving yield (Morinaka et al., 2006; Sakamoto et al., 2006). For example, the weakest allele of *OsBRI1* (*d61-7*), the counterpart of *Arabidopsis* BR receptor *BRI1*, produced erect leaf and generate greater biomass under high density planting conditions (Morinaka et al., 2006). The erect leaf trait of *OsDof12* overexpression lines is favorable for ideal plant architecture. However, similar to *d61-7* (Morinaka et al., 2006), at least under normal planting density, *OsDof12* overexpression lines would produce decreased spikelets. Therefore, whether the harvest index of *OsDof12* overexpression lines increase or not under different higher planting densities need to be further investigated.

Overexpression of *OsDof12* leads to shorter leaves and less grain yield under normal planting dense, which is unfavorable for utilization in plant breeding. On the contrary, whether the plants down-expressing *OsDof12* induce longer leaves and higher grain yield should be further validated. Indeed, we have tried in this direction but failed to obtain *OsDof12* suppressed lines with obviously distinguishable phenotypes from WT (Li et al., 2009b). We speculated this might result from functional redundancy in rice *Dof* genes, and the functional redundancy in *Dofs* have been demonstrated in Arabidopsis (Ahmad et al., 2013). Therefore, in our future study, we will try to better understand the full-version function of *OsDof12* by utilizing CRISPR/Cas9 (Belhaj et al., 2015) and Chimeric Repressor gene Silencing Technology (CRES-T) (Mitsuda et al., 2011).

In summary, we show the involvement of *OsDof12* in BR signaling coordination in rice and the effects of overexpression of *OsDof12* on rice plant architecture, and the findings imply *OsDof12* might be a potential genetic module for future rice breeding strategy.

## Author contributions

SL and LZ conceived and designed the experiments. QW and DaL performed most of the experiments. QW, DaL, SL, and LZ wrote the manuscript. DeL and XZ performed phenotypes observation and statistic analysis. XuL and XiL performed microscopic observation. All authors have read and approved the manuscript.

### Conflict of interest statement

The authors declare that the research was conducted in the absence of any commercial or financial relationships that could be construed as a potential conflict of interest.

## Supplementary material

Figure S1 **Comparison of the grain size between wild type and OD lines. (A)** There was no obvious difference between WT and *OsDof12* overexpression lines in grain size. Bar = 1.5 mm. **(B)** Statistical data of 1000-grain weight. Values are mean ± sd, *n* = 1000.

Figure S2 **Morphology and statistical analysis of the leaf length. (A)** Gross morphology of flag leaves, penultimate leaves and antepenultimate leaves. Bar = 8 cm. **(B)**The length of flag leaves, penultimate leaves and antepenultimate leaves in *OsDof12* overexpression plants was shorter than WT plants. Values are mean ± sd (*n* = 30). Single asterisk and double asterisks stand for *P* < 0.05 and *P* < 0.01 determined by student's *t*-test, respectively.

Figure S3 **Expression analysis of BR metabolism related genes in WT plants and transgenic plants overexpressing ***OsDof12*****. Expression level of *OsDof12* in OD plants was significantly higher than in WT plants. *D11* and *CYP734A6* was slightly but not obviously down-regulated in OD plants, while expression levels of the other BR metabolism related genes were comparable in both plants. n.d means not detected. Double asterisks stand for *P* < 0.01 determined by student's *t*-test.

Table S1 **Quantification of CS in WT and OD2**. Means ± SD of two replicates are shown (ng·g-1 F.W.).

Table S2 **The primers used for qRT-PCR in this study**.
