# Supplementary figures and images for: Overexpression of OsDof12 affects plant architecture in rice (Oryza sativa L.)
Source: Front Plant Sci. 2015 Oct 8;6:833. doi: 10.3389/fpls.2015.00833 (PMC4597119; doi:10.3389/fpls.2015.00833)

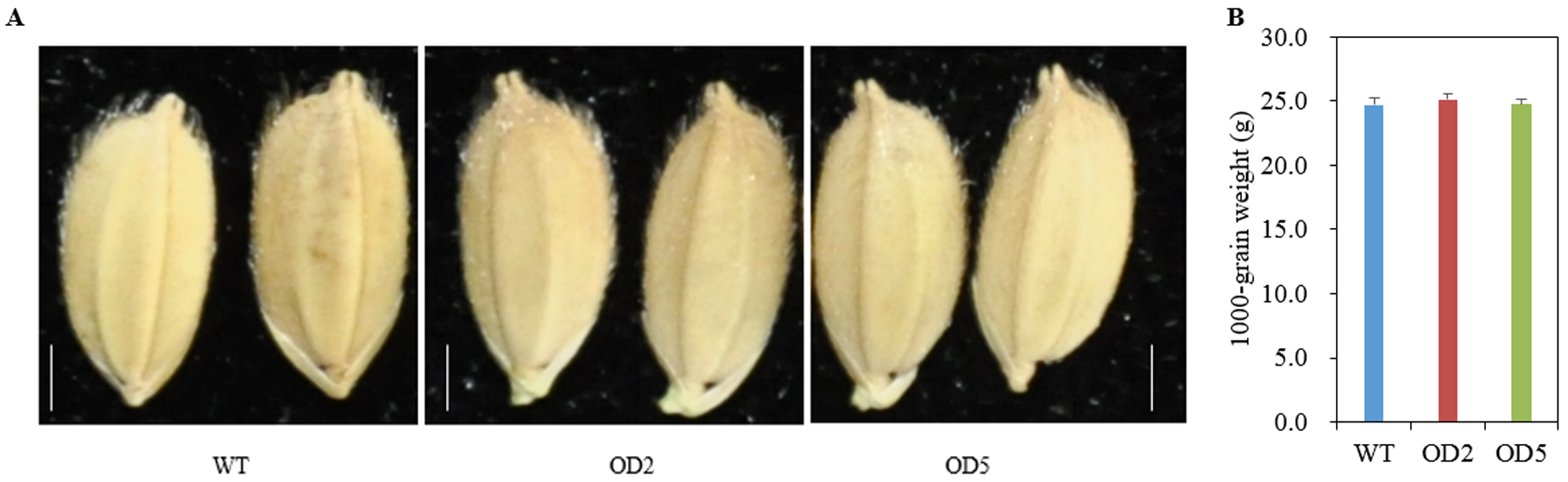

Supplement: Figure S1 — Comparison of the grain size between wild type and OD lines. (A) There was no obvious difference between WT and OsDof12 overexpression lines in grain size. Bar = 1.5 mm. (B) Statistical data of 1000-grain weight. Values are mean ± sd, n = 1000. [file Image1.TIF]

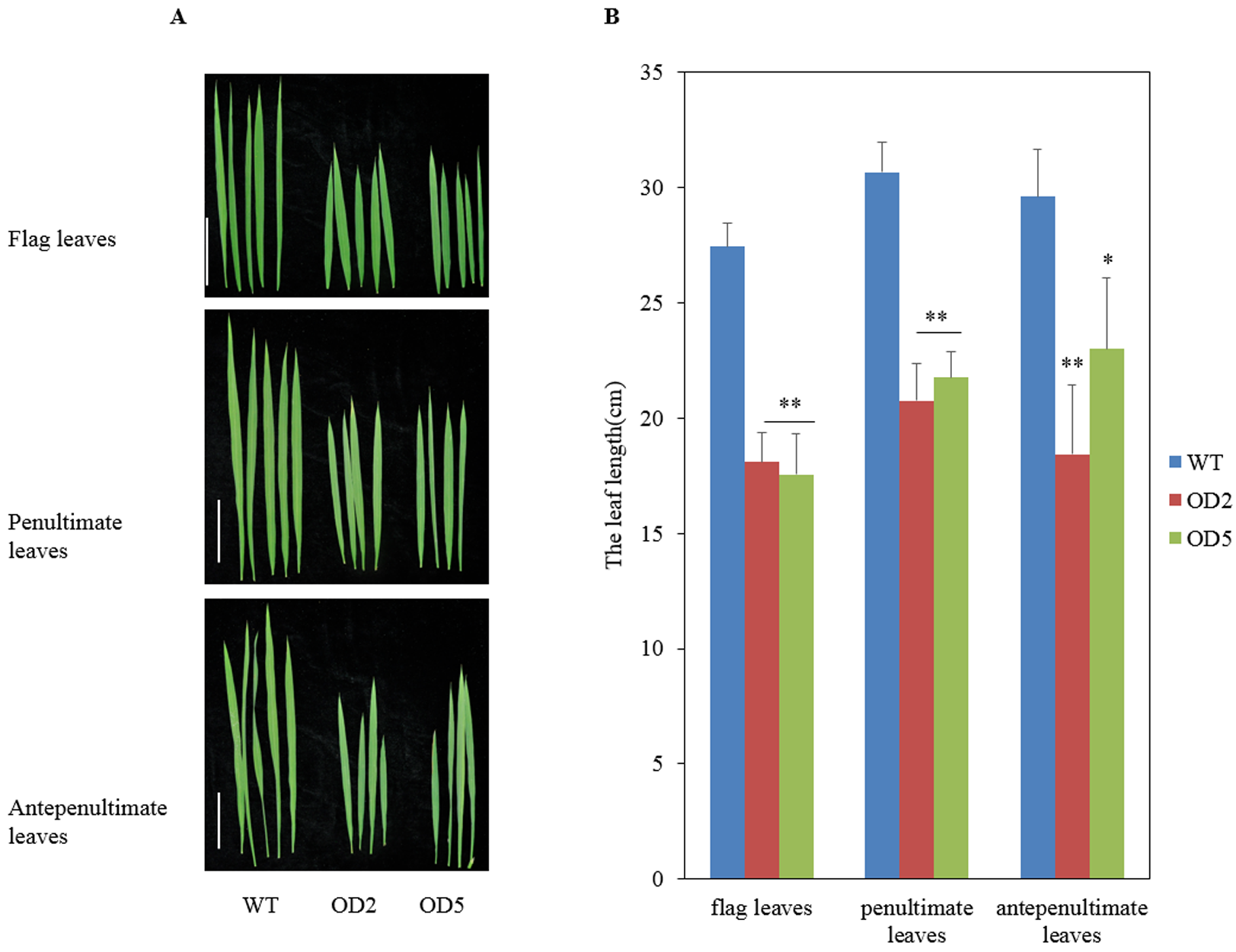

Supplement: Figure S2 — Morphology and statistical analysis of the leaf length. (A) Gross morphology of flag leaves, penultimate leaves and antepenultimate leaves. Bar = 8 cm. (B)The length of flag leaves, penultimate leaves and antepenultimate leaves in OsDof12 overexpression plants was shorter than WT plants. Values are mean ± sd (n = 30). Single asterisk and double asterisks stand for P < 0.05 and P < 0.01 determined by student's t-test, respectively. [file Image2.TIF]

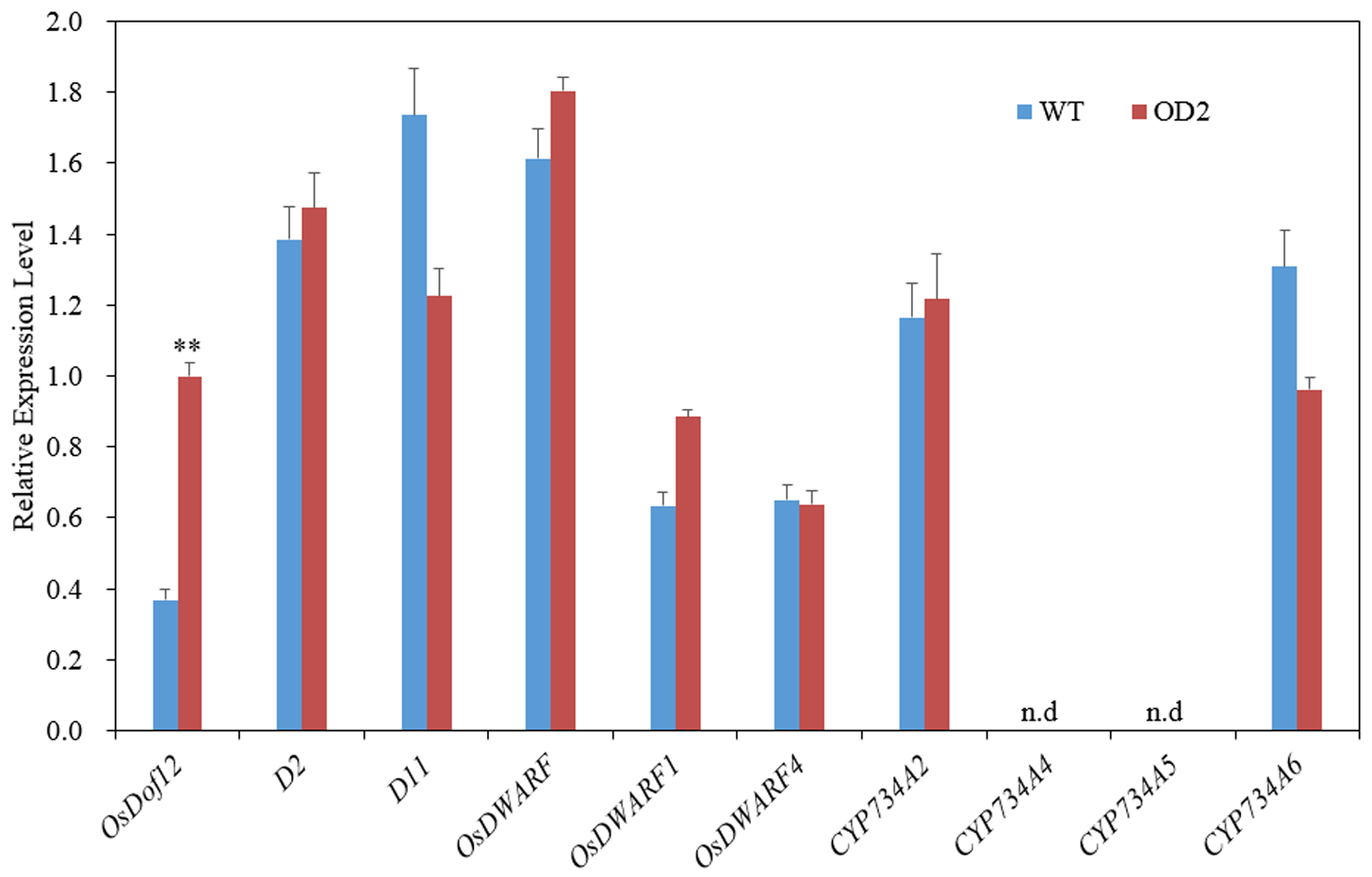

Supplement: Figure S3 — Expression analysis of BR metabolism related genes in WT plants and transgenic plants overexpressing OsDof12. Expression level of OsDof12 in OD plants was significantly higher than in WT plants. D11 and CYP734A6 was slightly but not obviously down-regulated in OD plants, while expression levels of the other BR metabolism related genes were comparable in both plants. n.d means not detected. Double asterisks stand for P < 0.01 determined by student's t-test. [file Image3.TIF]
