# Supplementary material for: Overexpression of OsDof12 affects plant architecture in rice (Oryza sativa L.)
Source: Front Plant Sci. 2015 Oct 8;6:833. doi: 10.3389/fpls.2015.00833 (PMC4597119; doi:10.3389/fpls.2015.00833)
Supplement: Table S1 — Quantification of CS in WT and OD2. Means ± SD of two replicates are shown (ng·g-1 F.W.). [file Table1.PDF]

Table S1 Quantification of CS in WT and OD2. Means ± SD of two replicates are shown (ng g^{-1} F.W.).

Analyte	CS
WT	0.13±0.01
OD2	0.13±0.02
