# Supplementary material for: Overexpression of OsDof12 affects plant architecture in rice (Oryza sativa L.)
Source: Front Plant Sci. 2015 Oct 8;6:833. doi: 10.3389/fpls.2015.00833 (PMC4597119; doi:10.3389/fpls.2015.00833)
Supplement: Table S2 — The primers used for qRT-PCR in this study. [file Table2.PDF]

Table S2 The primers used for qRT-PCR in this study.

Name<br>ID #	Forward(5'-3')	Reverse(5'-3')
UBQ<br>(Os03g13170)	AACCAGCTGAGGCCCAAGA	ACGATTGATTTAACCAGTCCA<br>TGA
OsDof12<br>(Os03g07360)	AGCTGCTGTTGCTGCATACT	ATGATGAAACCGATTGGGAT
OsBZR1<br>(Os07g39220)	CGTCGCCCACCTACAACCTC	TCGCCCAAATCGCAGCAT
DLT<br>(Os06g03710)	TGCGGATACTCAACGCCATCA	ACTCGCCGACTCCGGTGATC
OsBRI1<br>(Os01g52050)	CAGCTACTTGGCTATCTTGAAG<br>CTC	CCATTCTTGTTGAAGGTGTAC<br>TCCG
BU1<br>(Os06g12210)	GTAGCCAGCTTGATCTCATCTC	GGGACGACTCTACTGCATCA
OsLIC<br>(Os06g49080)	ATGCAGACAACAAATTTC	TTCCTCGAAGCTAATGTC
OsBAK1<br>(Os08g07760)	TCTTTGATGTGCCTGCTGA	TTTATTGCTGAAGGTATCTGTT<br>G
D2<br>(Os01g10040)	AGCTGCCTGGCACTAGGCTCTA<br>CAGA	ATGTTGTCGGAGATGAGCTCG<br>TCGGT
D11<br>(Os04g39430)	TTGGGTCATGGCATGGCAAGA<br>GCAA	TTGTTGCTGGAGCCAGCATTC<br>CTCC
OsDWARF<br>(Os03g40540)	ATGGTGTTGGTGGCGATTGGG<br>GTG	ATGTTGTTCCGCCCCAGGATG<br>TCC
OsDWARF1<br>(Os10g25780)	GACACTAGCTATGCCCAGATG	TGGTAGCTGTGGTTCTCAATC
OsDWARF4<br>(Os03g12660)	AGTCGCGTGCTGCCATTCTCGG<br>AG	AGCAAGCTCAGCAAGAGGTC<br>CAGG
CYP734A2<br>(Os02g11020)	TTTGTCCTAGACTGTGCCTTG	GTCTGATCCGGTCTGTTAAGG
CYP734A4<br>(Os06g39880)	GCCAAGAGACAACTAACAAAA<br>GG	AGTTGTGCTCTGATCGTACC
CYP734A5<br>(Os07g45290)	ACCTCCCACGACATCCTC	AAATGCATCGGACCGTAGAG
CYP734A6<br>(Os01g29150)	AAGATGAACCCGCTAACCAG	GCTACAACTTGGTCCTACGAT<br>C

Sequence data used in this study can be found in the rice genome annotation database

(http://rice.plantbiology.msu.edu).
